# A Preliminary Study of Quantitative MRI Cartilage Loss Fraction and Its Association With Future Arthroplasty Using the Osteoarthritis Initiative Database

**DOI:** 10.7759/cureus.64279

**Published:** 2024-07-10

**Authors:** Stephanie Jo, Ronnie A Sebro, Lei Zhang, Ze Wang, Linda Chang, Marc C Hochberg, Braxton D Mitchell

**Affiliations:** 1 Diagnostic Radiology and Nuclear Medicine, University of Maryland School of Medicine, Baltimore, USA; 2 Radiology, Mayo Clinic Jacksonville, Jacksonville, USA; 3 Medicine, University of Maryland School of Medicine, Baltimore, USA

**Keywords:** subchondral bone, articular cartilage, imaging biomarker, musculoskeletal mri, knee osteoarthritis/ koa

## Abstract

Background and objective

Osteoarthritis (OA) is the most common arthritis in the world. Despite the high disease burden, there is no therapy to prevent, halt, or reverse OA, and many clinical trials relied on radiographic biomarkers for therapy response. It is important to identify patients with early OA who will eventually need arthroplasty, the end-stage treatment for osteoarthritis. This pilot study evaluates a novel MRI biomarker, cartilage loss fraction, for association with future arthroplasty and evaluates its feasibility of use and effect size estimates.

Materials and methods

Publicly available knee MRIs from the Osteoarthritis Initiative were used. A total of 38 participants with Kellgren-Lawrence (K-L) grade >1 and 38 participants with K-L grade ≤ 1 at enrollment were matched in age, sex, race, and BMI, and assessed for the degree of full-thickness cartilage loss, or cartilage loss fraction. Univariate conditional logistic regression analysis was performed for differences in cartilage loss fractions between groups. Receiver operating characteristic (ROC) curve analysis was performed to assess the association of MRI biomarkers and knee arthroplasty during the eight-year follow-up.

Results

The medial femoral condyle, medial tibial plateau, total, and two-year progression cartilage loss fractions were significantly higher in participants with K-L grade >1 (p < 0.01 for all) and showed high area under the curve (AUC) values on ROC analysis (812, 0.827, 0.917, and 0.933, respectively). These results were comparable or more strongly associated with other OA grading schemes.

Conclusion

MRI biomarker cartilage loss fractions are significantly higher in subjects with K-L grade >1 and show a strong association with arthroplasty. After further validation, cartilage loss fracture may be used to predict future arthroplasty.

## Introduction

Osteoarthritis (OA) is the most common arthritis worldwide, affecting over 30 million people in the United States alone [1], and is one of the leading causes of disability along with diabetes and dementia [2]. Despite the high disease burden, there is limited treatment to halt, reverse, or prevent OA [3]. This is a missed opportunity for intervention, as OA has a relatively slow progression of disease over months to years.

With the exception of weight loss [4,5], no disease-modifying therapy is known to halt the progression of OA, and patients eventually need joint replacement. While the procedure is generally safe and effective, up to 3% of patients require critical care services after elective arthroplasty [6]. Additionally, complicated revision surgery may be necessary for up to 10-12% of patients within 10 years of hip or knee arthroplasty [7,8]. These statistics are especially problematic for younger patients, who have longer life expectancies and higher activity levels than older patients. At present, many clinical trials in search of OA disease-modifying therapy have been disappointing [9,10]. Much of this failure can be attributed to the heterogeneity of the disease and of the participants. To date, few useful subclassifications or biomarkers of early OA exist for targeted treatment [11]. 

Several imaging OA biomarkers are used for research, with limited clinical usage. For radiographs, Kellgren-Lawrence (K-L) grade [12] and minimal joint width [13] are commonly used. For MRI, several semi-quantitative scoring tools are available, including MRI OA Knee Score (MOAKS), which has very good to excellent reliability [14]. Several studies use machine learning to assess imaging features on three-dimensional (3D) MRI sequences [15-18]. 

However, these existing OA imaging biomarkers have limitations. While widely used in research, K-L grade has known disease heterogeneity [19] and joint space width measurement is highly dependent on standardized image acquisition [20]. Similarly, while accurate and reliable, semiquantitative knee OA scoring schemes on MRI are complex requiring multiple sequences and assessing multiple imaging findings [14]. 3D sequence-based machine learning algorithms may not perform as well on two-dimensional (2D) sequences, which are the clinical workhorses. Hence, a simpler, reliable MRI biomarker that can predict OA outcomes on a routine 2D clinical sequence is needed. We selected a standard 2D sagittal intermediate weighted fat suppression (Sag IW FS) sequence, which is commonly obtained in clinical practice, for image analysis. This pilot study evaluates a novel MRI biomarker, cartilage loss fraction, for association with future arthroplasty, and assesses the feasibility of performing these novel measurements and generating effect size estimates for a larger-scale effort. 

## Materials and methods

The Osteoarthritis Initiative (OAI) database

The OAI is a publicly available multicenter, longitudinal, prospective observational study of knee OA [21]. Longitudinal data collection over eight years was completed as of January 1, 2015. Relevant to this study, available data includes multi-year knee MRIs, radiographs, centrally scored K-L grade and MRI MOAKS, and arthroplasty status over the eight-year follow-up period. The International Workshop on Osteoarthritis Imaging (IWOAI) challenge best-performing algorithm using OAI MRI images was used to obtain cartilage volume [15] for comparison. OAI participants were assigned as progression subcohort if frequent knee symptoms in the past 12 months and radiographic tibiofemoral knee OA K-L grade greater than 1. Those who did not meet these criteria but with risk factors such as overweight/obesity, prior knee injury or surgery, family history, Heberden’s nodes, and repetitive knee bending were assigned incidence subcohort [22]. This study was reviewed by the University of Maryland Baltimore Institutional Review Board (HP-00107152) and was exempt.

MRI data and participant match

The analysis included 38 subjects in the progression subcohort and 38 subjects in the incidence subcohort, matched on age, sex, self-reported race, and BMI category. Baseline demographic variables are shown in Table 1.

**Table 1:** Participant characteristics *One subject identified as ‘Other’ and one subject identified as ‘African American’ in the progression subcohort were matched to ‘White’ subjects as no subject similar in age, sex, and BMI category in the incidence subcohort was available. OA: osteoarthritis; BMI: body mass index

Characteristics		Progression subcohort (n=38)	Incidence subcohort (n=38)
Age (years)	Median (range)	49 (45-51)	49 (45-51)
	Average (range)	48.3 (45-51)	48.3 (45-51)
Sex	Male, n (%)	19 (50%)	19 (50%)
	Female, n (%)	19 (50)	19 (50%)
Race^*^	White, n (%)	29 (76.3%)	31 (81.6%)
	African American, n (%)	8 (21%)	7 (18.4%)
	Other	1 (2.6%)	
BMI (kg/m^2^)	Median	31.5	30.7
	Average	31.7	30.6
	18.5 ≤ BMI <25, n (%)	0 (0%)	0 (0%)
	25≤ BMI <30, n (%)	5 (13.2%)	5 (13.2%)
	30≤ BMI <35, n (%)	16 (42.1%)	16 (42.1%)
	35≤ BMI <40, n (%)	15 (39.5%)	15 (39.5%)
	BMI ≥40, n (%)	2 (5.3%)	2 (5.3%)

MRI biomarker feature selection

With OA progression, there is a loss of extracellular matrix production and cartilage degradation, with the most severe form resulting in exposed subchondral bone. Subchondral bone is a dynamic adaptive tissue that provides mechanical support and nutritional supply for the overlying articular cartilage [23,24]. On imaging studies, full-thickness cartilage loss with exposed subchondral bone is associated with bone marrow edema-like lesions, subchondral cyst-like changes, and subchondral sclerosis. Given pathophysiological and imaging importance, we evaluated the degree of full-thickness cartilage loss with exposed subchondral bone (cartilage loss fraction) on MRIs as a potential OA biomarker.

MRI biomarker acquisition

The 2D Sag IW FS sequence had the following acquisition parameters: TE 30 msec, TR 3200 msec, flip angle 180 degrees, bandwidth 248 (Hz/pixel), and slice thickness 3 mm, x-resolution 0.357 mm, y-resolution 0.511 mm as indicated in OAI MRI protocol [25]. This sequence is commonly obtained in clinical MRI exams. From the baseline knee MRIs, the knee with more severe OA was selected (either left or right) for image analysis. 

Full-thickness cartilage loss is cartilage loss extending to the underlying subchondral bone with complete denudation. This was manually segmented on ImageJ (National Institutes of Health, Maryland, United States) and quantified in medial (medial femoral condyle, medial tibial plateau), lateral (lateral femoral condyle, lateral tibial plateau), and patellofemoral (patella, trochlea) compartments per clinical convention. The trochlea and femoral condyle articular surfaces were distinguished based on the condylopatellar sulcus, as this sulcus is clinically used for the evaluation of knee injury [26,27].

**Figure 1:**
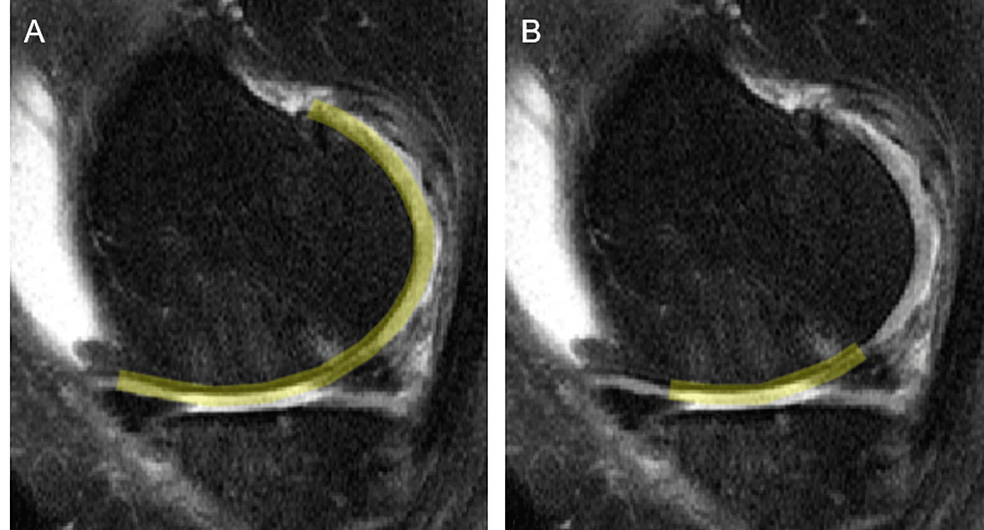
Example of cartilage loss fraction measurement on MRI. Sagittal IW FS sequence was used for the segmentation.  For each 2D image, completely denuded articular surface measurement (B, marked yellow) was divided by the subchondral bone measurement (A, marked yellow) and the full-thickness cartilage loss fraction was obtained. IW FS: intermediate-weighted fat saturation; 2D: two-dimensional

**Figure 2:**
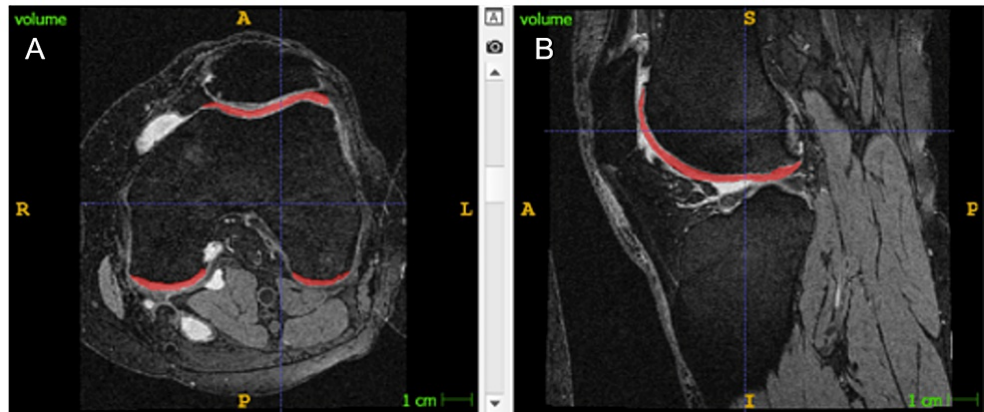
Example of IWOAI challenge best-performing algorithm cartilage segmentation. Automated femoral cartilage volume segmentation of 3D DESS sequence axial (A, cartilage marked red) and sagittal (B, cartilage marked red) reformats. IWOAI: International Workshop on Osteoarthritis Imaging; 3D: three dimensional; DESS: double echo steady state

To better compare the value among participants, the full-thickness cartilage loss was divided by the respective subchondral bone surface, normalizing the degree of cartilage loss for patient size and individual differences in anatomic morphology (cartilage loss fraction). The total cartilage loss fraction was obtained by dividing the sum of all cartilage losses by the sum of all subchondral bone surfaces. The progression of the 24-month total cartilage loss fraction compared to the baseline total cartilage loss fraction was obtained by subtraction of total cartilage loss fractions at two timepoints. For three participants, the 24-month follow-up MRI was not available, and the 12-month follow-up MRI was used instead. One participant was excluded from the two-year progression cartilage loss analysis since both 12-month and 24-month follow-up MRIs were missing. Figure 1 shows an example of cartilage loss fraction measurement. K-L grade and MOAKS from baseline imaging studies scored by the OAI central sites were used for analysis. Automated cartilage volumes of femoral cartilage, tibial cartilage, and patellar cartilage were obtained from IWOAI challenge best-performing algorithm [15] on the baseline and follow-up MRI images (Figure 2). 

Statistical analysis

First, we used conditional logistic regression analysis to compare imaging biomarkers (cartilage loss fractions, K-L grade, MOAKS, and automated cartilage volume) between the progression subcohort and the incidence subcohort. Second, we used receiver operating characteristic (ROC) analysis to evaluate the performance of imaging biomarkers in predicting knee arthroplasty, the treatment for end-stage OA, over the eight-year OAI follow-up period. Analyses were performed using the Rstudio version 4.3.1 software package (R Foundation for Statistical Computing, Vienna, Austria). 

## Results

Cartilage loss is greater in progression subcohort compared to incidence subcohort

**Figure 3:**
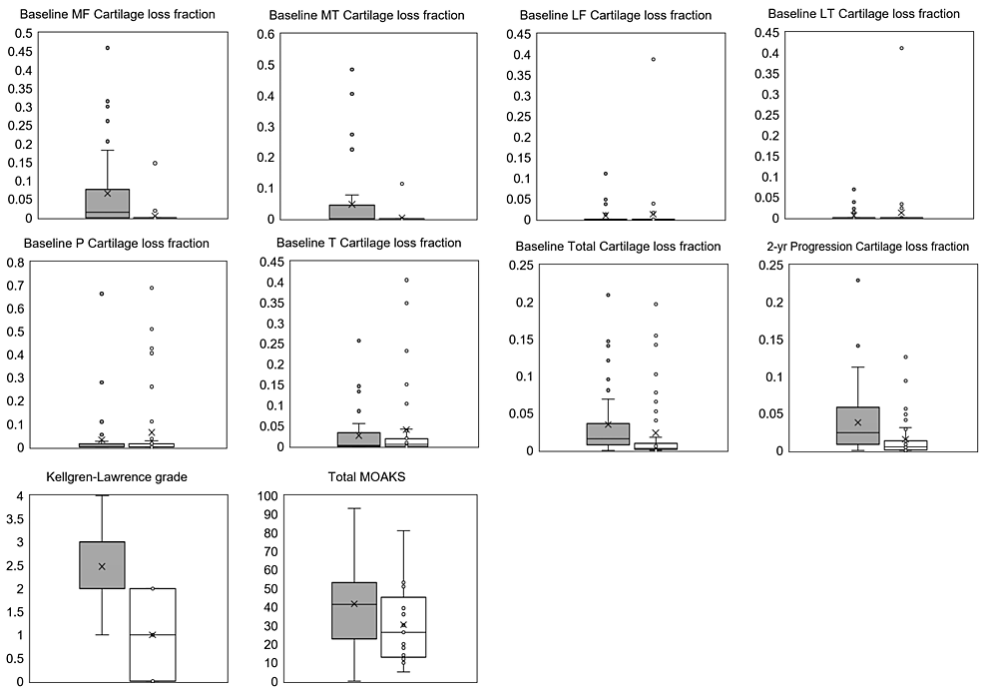
Box-Whisker plot of imaging biomarkers. Grey: Progression subcohort; White: Incidence subcohort MF: medial femoral condyle; MT: medial tibial plateau; LF: lateral femoral condyle; LT: lateral tibial plateau; P: patella; T: trochlea; MOAKS: MRI Osteoarthritis Knee Score

Conditional logistic regression analyses showed that mean medial femoral condyle, medial tibial plateau, total, and two-year progression cartilage loss fractions, K-L grade, and MOAKS were higher in the progression subcohort compared to incidence subcohort with a p-value equal to or less than 0.05 (Table 2 and Figure 3). This is in accordance with existing literature and supports that cartilage loss fraction can be an OA MRI biomarker on par with K-L grade and MOAKS. The mean lateral tibial plateau cartilage loss fraction was statistically significantly larger in the incidence subcohort, although given a single outlier (best seen in Figure 3) it is of unclear clinical significance. Mean trochlear and patellar cartilage loss fractions were higher in the incidence subcohort but not statistically different between the two subcohorts.

**Table 2:** Mean values (25th percentile-75th percentile) and conditional logistic regression p-values of imaging biomarkers. K-L: Kellgren-Lawrence; MOAKS: MRI Osteoarthritis Knee Score

Imaging biomarker	Progression subcohort (n=38)	Incidence subcohort (n=38)	p-value
Baseline medial femoral condyle cartilage loss fraction	6.7% (0-6.1%)	0.48% (0-0%)	<0.001
Baseline medial tibial plateau cartilage loss fraction	4.9% (0-4.5%)	0.30% (0-0%)	<0.01
Baseline lateral femoral condyle cartilage loss fraction	0.9% (0-0%)	1.3% (0-0%)	0.124
Baseline lateral tibial plateau cartilage loss fraction	0.5% (0-0%)	1.3% (0-0%)	0.03
Baseline trochlea cartilage loss fraction	2.6% (0-3.0%)	4.2% (0-1.9%)	0.875
Baseline patellar cartilage loss fraction	3.3% (0-1.2%)	6.7% (0-1.4%)	0.48
Baseline total cartilage loss fraction	3.5% (0.8-3.6%)	2.4% (0.1-0.94%)	<0.001
Two-year progression total cartilage loss fraction	3.9% (0.8-5.8%)	1.6% (0.02-1.4%)	<0.01
Baseline K-L grade	2.47 (2-3)	1.18 (0-2)	0.05
Baseline total MOAKS	41.7 (23.7-52.5)	30.5 (14-39.4)	<0.01

Cartilage loss fraction and knee arthroplasty have a strong association

**Figure 4:**
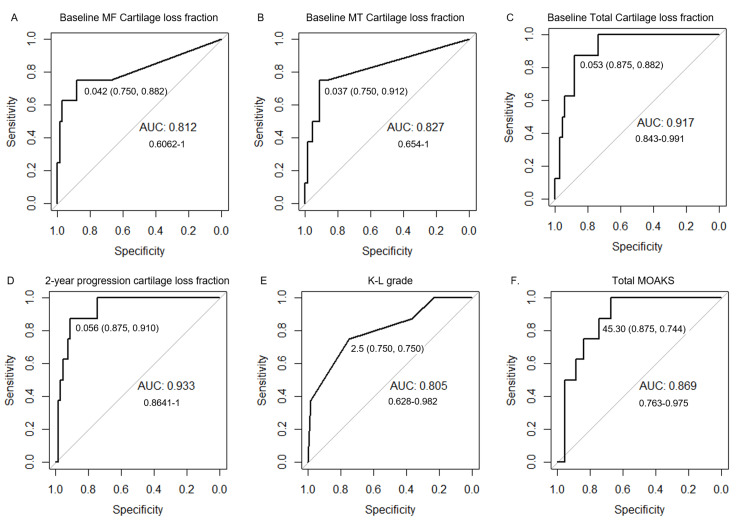
ROC analysis of 76 participants (38 incidence, 38 progression) matched in age, sex, race, and BMI category. Cartilage loss fractions of (A) baseline MF, (B) baseline MT, (C) baseline total, and (D) two-year progression were similar or more strongly associated with arthroplasty compared to (E) K-L grade, and (F) total MOAKS AUC value and 95% confidence intervals on graph, threshold value with sensitivity and specificity in parenthesis MF: medial femoral condyle; MT: medial tibial plateau; MOAKS: MRI Osteoarthritis Knee Score; ROC: receiver operating characteristic; BMI: body mass index; AUC: area under the curve; K-L: Kellgren-Lawrence

**Figure 5:**
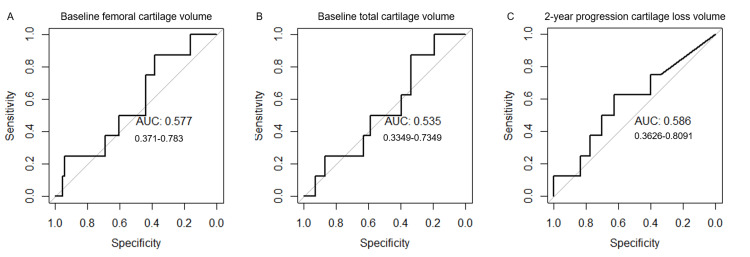
Automated machine learning algorithm quantification of cartilage volume did not show significant association with arthroplasty. AUC value and 95% confidence intervals on graph AUC: area under the curve

The eight-year incidence of knee arthroplasty was 10.3% (8/78); seven in the progression subcohort and one in the incidence subcohort. ROC analyses were performed to evaluate the performance of the imaging biomarkers in predicting knee arthroplasty. The area under the curve (AUC) was > 0.80 for baseline medial femoral condyle cartilage loss fraction (AUC 0.812, sensitivity 0.750, specificity 0.882) and baseline medial tibial plateau cartilage loss fraction (AUC 0.827, sensitivity 0.750, specificity 0.912). Baseline total cartilage loss fraction showed a stronger association with AUC of 0.917, sensitivity of 0.875, and specificity of 0.882. The strongest association with arthroplasty was seen with the two-year progression cartilage loss fraction with AUC of 0.933, sensitivity of 0.875, and specificity of 0.910. As expected of an established research tool, baseline MRI total MOAKS had a high association with arthroplasty with an AUC of 0.869, a sensitivity of 0.875, but a lower specificity of 0.744. Baseline radiographic K-L grade also showed similar AUC and sensitivity to medial compartment cartilage loss fractions of 0.805 and 0.750, but a lower specificity of 0.750. ROC curves and confidence intervals are shown in Figure 4. IWOAI algorithm baseline total, femoral, tibial, patellar, and two-year progression automated cartilage volumes were not significantly associated with arthroplasty (Figure 5; tibial and patellar cartilage data is not shown). 

## Discussion

OA is the most common arthritis and is one of the leading causes of disability in the United States. Given the limited disease-modifying treatment options available, exploring biomarkers that can predict end-stage OA at an early stage of the disease from routine patient care data is imperative. 

In this pilot nested case-control study, we compared a new MRI biomarker cartilage loss fraction obtained from commonly obtained 2D MRI images to established radiographic scoring system K-L grade, MRI semiquantitative MOAKS, and automated 3D sequence MRI cartilage volume quantification for their association with subsequent arthroplasty. Our preliminary results suggest that the cartilage loss fraction is a biomarker that is associated with future arthroplasty, similar or more strongly associated compared to commonly used imaging biomarkers. The MRI biomarker also corresponds with known important OA imaging features (full-thickness cartilage loss with exposed subchondral bone) and reflects known subchondral bone importance in OA pathophysiology [24]. Cartilage loss fraction is also a relatively simple MRI biomarker, which can be obtained from a single Sag IW FS sequence that is routinely obtained as part of the clinical knee MRI exam.

A consensus disease risk assessment system that is based on relevant pathophysiological findings and reliably associated with important clinical endpoints is critical for improved understanding and management of disease. Our pilot study suggests measuring cartilage loss fraction as a quantitative MRI biomarker is feasible and suggests a large effect size with future arthroplasty. While knee arthroplasty has excellent effectiveness and safety record, no surgery is completely free of risk and many patients with arthroplasty are outliving their prosthesis [8]. Further validation of the cartilage loss fraction evaluation with a larger sample size may be useful for identifying patients who need arthroplasty in the future as well as those who may not need it. Cartilage loss fraction can be obtained from a single sequence routine clinical MRI, which makes it more applicable to patients of all clinical settings and geography. 

This study has several limitations. First, we used data from relatively younger OAI participants (aged 45-51 years). We specifically explored these younger participants due to our interest in evaluating the new MRI biomarker in the younger age OAI participants who will eventually need arthroplasty. Young patients are more likely to outlive their prosthesis and will be affected by OA-induced disability for a longer period of time compared to older age patients. Second, we did not evaluate the pain and function of the patients in relation to the imaging biomarkers. The degree of disability from OA is often based on the severity of pain, which does not directly correlate with the severity of imaging (or structural) findings [28]. Third, we used MRI images from the OAI database. OAI MR images were obtained on 3.0 T MRI scanners with standardized protocols, with subjectively better image quality compared to routine clinical MRI exams obtained at the authors’ institutions. Hence, the results of the current study may have reduced applicability in routine clinical MRI knee exams, which include those from 1.5 T and even lower-strength open MRI magnets.

## Conclusions

Cartilage loss fraction is a quantitative MRI biomarker that appears strongly associated with future knee arthroplasty. Future studies will include older age participants, explore pain and function as OA outcomes, and design prospective studies with routine clinically obtained MRI exams to validate the current study. 
